# Comparing 3 Approaches for Making Vaccine Adoption Decisions in Thailand

**DOI:** 10.15171/ijhpm.2020.01

**Published:** 2020-01-20

**Authors:** Waranya Rattanavipapong, Ritika Kapoor, Yot Teerawattananon, Jos Luttjeboer, Siobhan Botwright, Rachel A. Archer, Birgitte Giersing, Raymond C. W. Hutubessy

**Affiliations:** ^1^Health Intervention and Technology Assessment Program (HITAP), Ministry of Public Health, Nonthaburi, Thailand.; ^2^Saw Swee Hock School of Public Health, National University of Singapore, Singapore, Singapore.; ^3^Asc Academics, Groningen, The Netherlands.; ^4^World Health Organization (WHO), Genève, Switzerland.

**Keywords:** Vaccine, Universal Health Coverage, Health Technology Assessment, Priority Setting, Thailand

## Abstract

**Background:** The World Health Organization (WHO) has developed the Total System Effectiveness (TSE) framework to assist national policy-makers in prioritizing vaccines. The pilot was launched in Thailand to explore the potential use of TSE in a country with established governance structures and accountable decision-making processes for immunization policy. While the existing literature informs vaccine adoption decisions in GAVI-eligible countries, this study attempts to address a gap in the literature by examining the policy process of a non-GAVI eligible country.

**Methods:** A rotavirus vaccine (RVV) test case was used to compare the decision criteria made by the existing processes (Expanded Program on Immunization [EPI], and National List of Essential Medicines [NLEM]) for vaccine prioritization and the TSE-pilot model, using Thailand specific data.

**Results:** The existing decision-making processes in Thailand and TSE were found to offer similar recommendations on the selection of a RVV product.

**Conclusion:** The authors believe that TSE can provide a well-reasoned and step by step approach for countries, especially low- and middle-income countries (LMICs), to develop a systematic and transparent decision-making process for immunization policy.

## Background

Traditionally, ministries or departments of health had sole responsibility for health service policy at the national level. These entities oversaw agenda setting, policy formulation and policy implementation. However, this trend has changed rapidly in recent years with the rise of universal health coverage (UHC); many countries have established new powerhouses for their UHC management. For example, Social Insurance Administration Organization in Indonesia, National Health Agency in India, PhilHealth in the Philippines, and Vietnam Social Security Office in Vietnam.

These institutional changes introduce new challenges for vaccine policy, since the responsibility for vaccine priority setting is often shared by multiple bodies which may have different interests. These challenges are in addition to other contextual and process factors that limit national priority setting for vaccines in low- and middle-income countries (LMICs), including political power, financial constraints, the influence of donor and industry priorities, a lack of explicit frameworks and participation of key stakeholders, and the inappropriate use of priority setting criteria.^1-5^ One way of addressing some of the above mentioned challenges is to develop a framework for supporting successful priority setting. To this end we piloted a new priority setting framework for vaccines, Total System Effectiveness (TSE), which has been renamed to Capacity-led Assessment for Priority-setting of Immunization, in Thailand, where the vertical vaccination program, previously part of the Ministry of Public Health (MOPH), has been reclassified to be part of the newly established and autonomous public health authority called the National Health Security Office (NHSO).

The Thai National Expanded Program on Immunization (EPI) was first introduced in 1977.^6^ Since then, priority vaccines have been provided free of charge to all the eligible population at public healthcare facilities in Thailand. The EPI currently covers 9 vaccine preventable diseases, namely tuberculosis, hepatitis B, diphtheria, tetanus, pertussis, poliomyelitis, measles, mumps, rubella, Japanese encephalitis, influenza, and cervical cancer.^7^

Previously, vaccine prioritization and selection were the joint responsibility of the MOPH and the Advisory Committee on Immunization Practices (ACIP). The ACIP is a subcommittee under the National Vaccine Committee and forms the National Immunization Technical Advisory Group for Thailand. It was established with a mandate to propose priority vaccines to the National Vaccine Committee and formulate recommendations for new vaccine introductions into EPI. The ACIP committee consists of experts in various disciplines including vaccinology, immunology, virology, infectious diseases, epidemiology, public health, and preventive medicine, and is chaired by the Director General of the Department of Disease Control (DDC) under the MOPH. Before 2002, the DDC acted as a vertical program manager in charge of the selection, procurement and management of the EPI program and vaccine management within MOPH.^6^

Since the establishment of the UHC scheme in 2002, which currently covers 75% of the Thai population, the NHSO has held the UHC budget independently from the MOPH.^8^ It has been responsible for the financial management of all medicines used under the UHC scheme, in addition to all vaccines used for entire population in the country. This has incited a new mechanism for vaccine prioritization and selection and altered the roles of ACIP and DDC/MOPH. The NHSO reference the National List of Essential Medicines (NLEM) as its pharmaceutical benefit package. The NLEM subcommittee, under the National Drug Committee (developed in 1981), is currently the decision-making body for selecting medicines and vaccines for the NLEM. The ACIP has shifted into an advisory role upon the creation of the subcommittee.

Having 2 separate systems for vaccine product prioritization can trigger disagreements on policy decisions regarding vaccine adoption by the UHC manager or its reference, ie, the NLEM subcommittee in Thailand. For example, human papillomavirus vaccine (HPV) was proposed by the ACIP long before its implementation by the NHSO in 2017 and other new vaccines such as Rotavirus vaccine (RVV) and pneumococcal conjugated vaccine (PCV) recommended by ACIP, are yet to be implemented by the NHSO. More detailed information on the ACIP and HPV case study is available in the literature published elsewhere.^7,9,10^

Misalignment between the vaccine advisory group and the policy-making body responsible for UHC benefit package development may be inevitable if the 2 adopt different perspectives on vaccine prioritization. Often, the technical advisory group on vaccines focus on safety and efficacy/effectiveness of vaccine introduction, given the group is mostly comprised of clinicians and are less fixated on the financial and other political pressures in issuing a new vaccination policy. In contrary, the UHC benefit package committee is by nature concerned with implementation and financial sustainability.

Priority-setting processes have been introduced to guide the reimbursement decisions, and health technology assessment (HTA) has been proposed as a priority-setting tool.^1,11,12^ HTA is defined as “the systematic evaluation of the properties and effects of a health technology, addressing the direct and intended effects of this technology, as well as its indirect and unintended consequences, and aimed mainly at informing decision-making regarding health technologies.”^13^ HTA has proven to help decision-makers improve the efficiency of resource allocation in Thailand.^1,11,12,14^

In 2018, the Health Intervention and Technology Assessment Program (HITAP) and the World Health Organization (WHO) undertook a pilot project to understand whether TSE approach could support the harmonization of the vaccine policy process in Thailand. TSE is a concept which primarily aims to strengthen country frameworks for evaluating vaccine products, as well as communicating the priorities expressed by countries to inform regional and global discussions on vaccine preferences. The TSE framework articulates the steps for a structured, systematic and transparent product selection process, based on multi-criteria decision analysis (MCDA).^15^

The TSE pilot project was conducted in Indonesia, Mali, Thailand, and Zambia as an exploratory study to assess the feasibility and usefulness of the TSE approach to inform vaccine policy. Thailand was chosen to represent the pilot country with well-established priority setting processes and to compare TSE with such existing mechanisms. Using the MCDA approach,^16^ in Thailand’s pilot, TSE model was designed as a practical tool run on Excel^®^ worksheets for supporting the development of immunization policy. TSE allowed local inputs to populate the model and in line with MCDA methodology, it scored vaccine performance against multi decision criteria.

The objectives of this paper are firstly to review the decision-making criteria and respective processes of ACIP and the subcommittee of NLEM in vaccine prioritization. Second, to explore whether TSE is a useful approach that can reconcile the 2 current mechanisms in Thailand, by comparing the TSE outcomes with those of ACIP and the subcommittee of NLEM. Lastly, to discuss whether the lessons from “one country, two systems experience” in Thailand can be useful for other countries where the national immunization program are or will become part of the UHC benefit package in the future. The authors will conclude by discussing the potential applications of their findings to other LMIC settings.

## Methods

The TSE approach was piloted in Thailand, starting from April to December 2018. For the first objective, authors conducted document reviews of the term of reference for the ACIP and NLEM subcommittee members as well as other publicly available government documents,^17,18^ interviews with Secretariat of the ACIP and NLEM (1 person of each Secretariat team), group discussions through key vaccine-related stakeholders meetings for which their minutes are available via this link http://www.globalhitap.net/resources/reports-publications-2/,^19,20^ and had direct observation of the meeting between the ACIP and NLEM on the RVV to understand the processes of the ACIP and NLEM subcommittee with regard to vaccine policy decisions. The documents review, the interview with Secretariat of the ACIP and NLEM, and the discussion during the stakeholder meetings were mainly centred on the overall governance, decision-making processes and criteria used for the vaccine prioritization and selection. Open-ended questions were deployed to collect qualitative information and provoke discussions in the stakeholder meeting on the points mentioned above. The discussions were translated into the narrative and figures/tables presented in the results section.

In order to consider the utility of the TSE approach in Thailand, the authors focused on the process-based approach, and used a one-vaccine case study of various RVV products employing TSE and both the ACIP and NLEM subcommittee methodology. Five hypothetical RVV product profiles were developed for the exercise and an excel-based MCDA model was developed specifically for the TSE-based RVV selection in Thailand (TSE pilot), incorporating parts of existing models including UNIVAC,^21^ V-TIA,^22^ and C4P.^23^ This TSE model comprised of 3 main components: decision criteria, estimation of the vaccine performance based on decision criteria, and a scoring and ranking process.

Criteria to be included in the TSE model was decided during the first stakeholder meeting convened in May 2018 with representatives from the NLEM subcommittee and ACIP, relevant ministry departments including DDC, health insurance, academicians, and manufacturers.^19^ Stakeholders were asked to complete an open-ended questionnaire stating their most important criteria for selecting a vaccine product in the Thai context and then to subsequently rank the criteria. Due to time constraints, inputs from 15 respondents were collected and the top 5 criteria were used as the decision criteria for the RVV selection model as decided by the stakeholders involved in the TSE pilot study.

Thailand-specific input parameters were computed to make the model relevant locally and to generate the performance scores for the different vaccine products. Parameters inputs included epidemiological data (eg, birth cohort, disease burden and numbers of outpatient and hospitalization), vaccine characteristics (eg, vaccine efficacy and vaccination schedule), cost data and other parameters such as coverage and socio-economic status (Supplementary file 1, Table S1). Parameters were generated from government reports, published literature, and expert opinions. Vaccine characteristics of the 5 hypothetical vaccine products were modified to reflect the Thai context (Supplementary file 1, Table S2). The detailed methodology for estimating the vaccine performance based on decision criteria are shown in Supplementary file 2. 1 USD was considered equivalent to 33.25 baht as per the exchange rates on July 2, 2018 (https://www.bangkokbank.com/). The threshold for the incremental cost-effectiveness ratio as current used in Thailand was set at 160 000 THB per disability-adjusted life year averted.^24^

Scoring was done by giving 100 points to the best performing vaccine and 0 points to the least performing vaccine products. Aggregated scores of the vaccine products were generated by linking the scores on different criteria with the criteria weights. Within MCDA methodology, weights represent the value stakeholders place on the different decision criteria. Given that the pilot study could only reach out to a limited number of stakeholders for criteria selection, equal weights were assigned to all the decision criteria.

In the second stakeholder meeting conducted in August 2018 in Thailand, the TSE findings were presented against the decision-making criteria set by the ACIP and NLEM subcommittee, as well as the TSE stakeholders (as a result of the first meeting).^20^

## Results

Process of Vaccine Prioritization and Selection in Thailand

**Figure 1:**
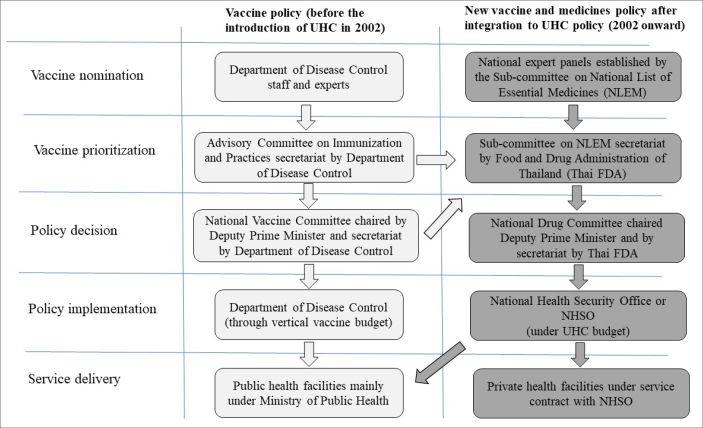
The National Vaccine Policy in Thailand Before and After UHC Introduction in 2002. Abbreviation: UHC, universal health coverage.

The national vaccine policy in Thailand before and after UHC introduction in 2002 is depicted in Figure 1. The ACIP’s role remains the same in providing a recommendation on vaccine and immunization policy formation; however, the request needs to be passed to the subcommittee of the NLEM for the inclusion of new vaccines in the reimbursement list. For the vaccines not included in the NLEM vaccines list, individuals must bear the cost of the vaccines themselves.

MCDA has been applied to the process of vaccine prioritization and selection by both the ACIP and the subcommittee of the NLEM. The common criteria considered by both the ACIP and subcommittee of the NLEM are: disease burden, disease prevalence, vaccine safety and efficacy, and estimated budget. In addition to these common criteria, the ACIP also considers local vaccine production capacity while the subcommittee of the NLEM considers cost-effectiveness and equity. For vaccines considered by the NLEM, the ACIP serves as a platform for information exchange, and submits evidence on disease prevalence, diseases burden, safety and efficacy of vaccine to the NLEM subcommittee as inputs for their consideration. The ACIP recommendations typically focus on a list of vaccine types, eg, HPV vaccine, PCV, ranked by their preference order. However, the ACIP do not specify the branding of vaccine products to be included.

The subcommittee of the NLEM, with support from Health Economic Working Group, then produces local evidence on cost-effectiveness and budget impact analysis to feed into the decision-making,^8^ ensuring the efficient allocation of resources and financial sustainability for the UHC payer. Like other medicines, NLEM adopts a “choose one policy” which translates as only one specific type of vaccine product/ brand being listed together with their maximum procurement price to inform procurement agencies eg, NHSO or hospitals.

Similarity and Differences in Between TSE and Existing Processes Used by Both Committees – The ACIP and the Subcommittee of the NLEM

Similar to the ACIP and the subcommittee of the NLEM, TSE uses an MCDA approach. However, while the ACIP and NLEM have fixed criteria to consider, the criteria for TSE can be flexible based on the decision question at hand. The output from the quantitative stage of TSE provides a ranking of available options, as is with the ACIP process.

The top 5 criteria identified for the TSE approach, as ranked by stakeholders through the questionnaires, were found to be as follows: safety, health impact, budget impact, delivery costs and cost effectiveness. Safety, health impact and budget impact were ranked by 85%-93% of stakeholders to be among the top 5 criteria (Table 1). Table 2 summarizes the criteria used by the 3 different decision-making bodies.

**Table 1:** Top Decision Criteria Based on Rankings in a Local Stakeholder Meeting

**Healthcare Priority**	**Working Definition Used in RVV Selection Model (TSE-Pilot)**	**Number of Votes**
Health impact/effectiveness/efficacy	Rotavirus cases averted with vaccination	15
Safety	Number of Intussusception hospitalizations due to vaccine	14
Budget impact	Overall 5-year budget impact including the cost of program	13
Delivery costs	Transport and storage costs for vaccines	7
Cost-effectiveness	Incremental costs per DALY saved	3
Burden of disease	Population size affected by the disease	3
Access to vaccine	Availability of the vaccine	2
Vaccine security	Ease of vaccine procurement and production security	2
Equity	Coverage across different society strata by income quintiles	1

Abbreviations: DALY, disability-adjusted life year; TSE, Total System Effectiveness; RVV, rotavirus vaccine.

**Table 2:** Criteria Considered in the 3 Decision-Making Approaches for Vaccine Product Prioritization

**Criterion**	**Considered by ACIP**	**Considered by NLEM**	**Identified by Stakeholders Through TSE Approach**
Disease burden	Yes	Yes	Yes (under ‘health impact’)
Disease prevalence	Yes	Yes	Yes (under ‘health impact’)
Vaccine efficacy	Yes	Yes	Yes (under ‘health impact’)
Vaccine safety	Yes	Yes	Yes
Budget impact	Yes	Yes	Yes
Cost-effectiveness	No	Yes	Yes
Capacity for local manufacturing	Yes	No	No
Equity	No	Yes	No
Delivery cost	No	No	Yes

Abbreviations: ACIP, Advisory Committee on Immunization Practices; NLEM, National List of Essential Medicine; TSE, Total System Effectiveness.

**Figure 2:**
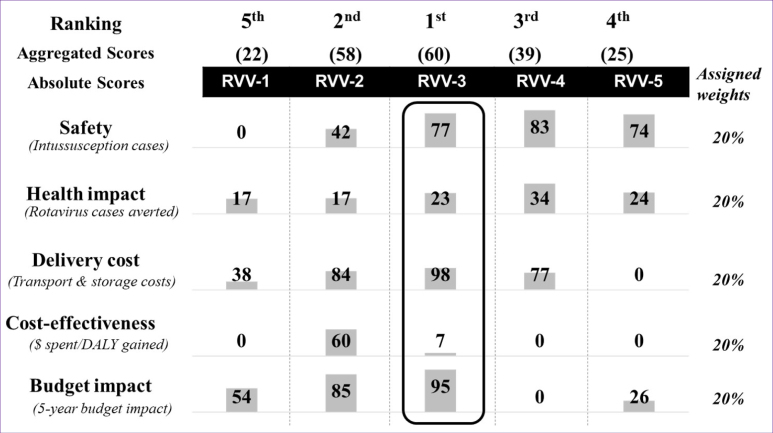
Ranking of the 5 Hypothetical RVVs From the RVV Selection Model (TSE-Pilot). Abbreviations: DALY, disability-adjusted life year; TSE, Total System Effectiveness; RVV, rotavirus vaccine.

When assessing the different types of RVV against the base case parameters, no vaccine product performed the best against all 5 criteria. The RVV selection model (TSE-pilot) found that RVV-3 was identified as the top-ranking vaccine, followed by RVV-2 and RVV-4 (Figure 2).

Table 3 shows the quantitative results for the top ranked vaccines using the RVV selection model (results are elaborated in Supplementary file 1, Table S3).

**Table 3:** Results From RVV Selection Model (TSE-Pilot) for the Top 5 Criteria Ranked by Thai Stakeholders

**Base-Case**		**‘No Vaccine’ Scenario**				
Size of birth cohort		679 502				
Total number of cases from 0-59 months		337 596				
Total hospitalizations		50 054				
Total deaths		74				
Total healthcare cost spent		6.77 million USD				
**Decision Criteria**	**Outcome Measures**	**RVV-3** **(Rank 1)**	**RVV-2** **(Rank 2)**	**RVV-4** **(Rank 3)**	**RVV-5** **(Rank 4)**	**RVV-1** **(Rank 5)**
Safety	Number of intussusception cases	4	11	3	5	19
Health impact	Number of cases averted	77 776	58 265	114 295	80 984	56 239
Delivery costs	Delivery and storage costs (USD)	8102	47 142	123 584	534 635	332 529
Cost-effectiveness	ICER in comparison to no vaccine (USD/DALY averted)	1254	1866	6830	7125	7113
Budget impact	5-year budget impact (million USD)	8.83	9.57	65.76	48.55	30.37

Abbreviations: RVV, rotavirus vaccine; TSE, Total System Effectiveness; DALY, disability-adjusted life year; ICER, incremental cost-effectiveness ratio.

When comparing the results of running the RVV selection test case using the ACIP, NLEM, and TSE approach, all 3 reached similar conclusions (Table 4).

**Table 4:** Ranking of the 5 Hypothetical RVVs Comparing 3 Priority Setting Tools, ie, TSE, ACIP and NLEM

**Vaccine Products**	**Priority Setting Tools**		
	**ACIP**	**NLEM** ^a^	**TSE**
RVV-1	Rank 3	-	Rank 5
RVV-2	Rank 2	-	Rank 2
RVV-3	Rank 1	Rank 1	Rank 1
RVV-4	Rank 3	-	Rank 3
RVV-5	Rank 3	-	Rank 4

Abbreviations: RVV, rotavirus vaccine; TSE, Total System Effectiveness; ACIP, Advisory Committee on Immunization Practices; NLEM, National List of Essential Medicines. ^a^NLEM applies ‘choose one policy,’ it does not generate ranks for all the vaccine products.

To compare the TSE-pilot, with the existing criteria in Thailand, the scores and rankings of the 5 hypothetical RVVs were evaluated against the ACIP and the subcommittee of the NLEM criteria (Table 5). Thailand’s established priority setting mechanisms, the NLEM and ACIP, offered RVV-3 as the optimal choice. In the case of the NLEM process, where only one product can be selected in its “choose one policy,” RVV-3 would be added to the benefits package. For the ACIP, the results indicate that RVV-3 would be ranked first, RVV-2 would be ranked second and RVV-1, 4, and 5 would be ranked third.

**Table 5:** Results of the 5 Hypothetical RVVs Used in the RVV Selection Model (TSE-Pilot) in Thailand, Against the ACIP and NLEM Criteria

**Criteria**	**Outputs From RVV Selection Model (TSE-Pilot)**	**Scoring**				
		**RVV-1**	**RVV-2**	**RVV-3**	**RVV-4**	**RVV-5**
**Criteria by ACIP**						
1. Disease prevalence (size of population affected)	337 596	5	5	5	5	5
2.Disease burden (case fatality rate)	0.02%	1	1	1	1	1
3.Vaccine effectiveness	Total case averted No vaccine = 337 596 RVV-1 = 56 239 (16.7%) RVV-2 = 58 265 (17.3%) RVV-3 = 77 776 (23.0%) RVV-4 = 114 295 (33.9%) RVV-5 = 80 984 (24.0%)	1	1	1	1	1
4.Vaccine safety	Incidence of intussusception cases (1-7 days risk period), per 100 000 per year RVV-1 = 19 (0.02%) RVV-2 = 11 (0.01%) RVV-3 = 4 (0.004%) RVV-4 = 3 (0.003%) RVV-5 = 5 (0.005%)	4	4	5	5	5
5. Budget impact	5-year budget impact (THB) RVV-1 = 1033 million RVV-2 = 325 million RVV-3 = 105 million RVV-4 = 2236 million RVV-5 = 1651 million	2	4	5	1	1
6. Vaccine production in country	NA	Hypothetical RVV				
**Total scores (out of 25)**		**13**	**15**	**17**	**13**	**13**
**NLEM’s Additional Criteria to ACIP** ^a^						
1. Cost-effectiveness analysis	ICER, compared to ‘No vaccine’ scenario (THB per DALY loss averted) RVV-1 = 242 000 THB RVV-2 = 63 500 THB RVV-3 = 12 000 THB RVV-4 = 232 300 THB RVV-5 = 242 200 THB	Dominated	Dominated	12 000	699 900 (compared to RRV-3)	Dominated
2. Budget impact	5-year budget impact (THB) RVV-1 = 1033 million RVV-2 = 325 million RVV-3 = 105 million RVV-4 = 2236 million RVV-5 = 1651 million	1033 million	325 million	105 million	2236 million	1651 million
3. Equity across health problems	NA	Hypothetical RVV				

Abbreviations: ACIP, Advisory Committee on Immunization Practices; ICER, incremental cost-effectiveness ratio; NA, not applicable; NLEM, National List of Essential Medicines; RVV, rotavirus vaccine; THB, Thai Baht; TSE, Total System Effectiveness; DALY, disability-adjusted life year. ^a^NLEM applies ‘choose one policy,’ it does not generate ranks for all the vaccine products.

As detailed in Table 4, the ACIP criteria ranked the bottom 3 vaccine products with an equivalent score at rank 3, whereas the RVV selection model (TSE-pilot) was able to discriminate between the 3 vaccine products with its scoring and ranking methodology.

## Discussion

To our knowledge, this is the first study examining the application of the TSE concept for vaccine introduction in LMICs. The WHO is piloting TSE as an approach for promoting evidence-based, demand driven vaccine selection, in line with existing country policy processes. Given that TSE allows multiple decision-making criteria to be incorporated in a transparent and systematic manner, it may be able to help reconcile concerns currently faced by different decision-making bodies in a country with 2 separate public health authorities responsible for policy development and holding technical advisory roles, as is the case with ACIP and NLEM subcommittee in Thailand.

We found that the 3 priority setting approaches, ie, the ACIP’s, NLEM’s and TSE offered the same policy choice. This may be explained by the fact that all the 3 approaches are based on MCDA with a similar qualitative aspect, use of quantitative evidence and had some overlapping criteria (ie, cost and outcomes/effectiveness data related to vaccine use). Although the top ranked vaccine product may not have performed best on all relevant criteria and is also not the cheapest vaccine option, it emerges as an optimal choice based on the MCDA used by all 3 approaches.

A major difference between the 3 processes is the mechanism by which criteria is selected and included. In the TSE framework, criteria are chosen according to the decision question at hand, whereas in ACIP and subcommittee of NLEM deliberations, the criteria is already articulated.^1,8^ Since there are well-established decision-making frameworks and governance in Thailand, such flexibility may not be necessary. However, when considering TSE at the global level, this flexibility to select the criteria based on decision question can allow for better discrimination between options. This would encourage countries to identify the decision criteria that is locally relevant, and may prompt the consideration of a broader set of criteria than the scientific considerations commonly considered by National Immunization Technical Advisory Groups.

Differences in perspectives and definitions of the criteria adopted in the 3 approaches were observed. For example, the ACIP includes disease burden in the way of measuring health impact and measuring case fatality rate whereas the RVV selection model (TSE-pilot) captures rotavirus cases averted as an indicator to account for the health impact. In addition, the application of the ACIP and NLEM criteria to different products of the same vaccine may not be able to differentiate if based on the epidemiological criteria eg, disease prevalence and burden. TSE can be seen as having an advantage over the ACIP mechanism as it is able to make a stronger differentiation between products of the same vaccine type. TSE can be designed to include a wide range of criteria for a one-vaccine evaluation. Whereas the ACIP mechanism has been established with the purpose of evaluating across vaccine types, with a strong focus on clinical aspects. Thus, in this study, while TSE could identify differences in each products performance in preventing rotavirus cases, ACIP ranked all 5 products the same in terms of disease burden and prevalence.

During the first stakeholder meeting, the chair of the subcommittee of the NLEM expressed the importance of decision-making bodies on health intervention and technology adoption being independent and policy neutral.^19^ They highlighted the value in the ACIP and the subcommittee of the NLEM fulfilling different functions as it enables the 2 committees to hold each other account. In the current status quo, the ACIP make an initial shortlist based predominantly on public health benefit, then the subcommittee of NLEM considers whether there is an economic argument for adding the recommended vaccine to the benefits package, in view of health interventions more broadly. Thus creating a check-balance mechanism that ensures that the introduction of a new and high-cost vaccine to the national reimbursement list is cost-effective, sustainable and affordable for the country. Nonetheless, the exercise of utilizing the TSE framework jointly with ACIP and NLEM could encourage a platform for deliberation in terms of the stakeholders, criteria, and the evidence involved in each process and could bring about better alignment between the 2. This may be true for other countries in which the immunization and broader UHC decision-making processes or financing streams are not combined. TSE may thus be able to help reconcile different interests and concerns regarding vaccine introduction policy between different priority setting agencies in UHC countries.

This study highlighted that, if used in real policy, the TSE approach may bring about a significant shift in the political and economic aspects of vaccine decision-making in Thailand. Given that TSE requires technical expertise in economics and modelling, individual with these skills set eg, health economists, analysts, technocrats would need to play a more active role in advising policy than in the current system at the ACIP, which focuses predominantly on the clinical aspects of the vaccine. Although the NLEM applies health economic evidence alongside clinical information, there is a deliberative process that allows qualitative data (such as urgency and equity concerns) to be included in the decision-making process. The quantitative TSE model was more rigid in this sense, though it can make vaccine decisions more transparent and consistent.

There are some limitations in the study design. While the RVV selection model developed to reflect the TSE approach provided the flexibility to incorporate country priority issues and generate good quality quantitative evidence, it required locally relevant specific inputs (parameters). Precision decisions depends on an accuracy and quality of data which can be difficult for other LMIC settings faced with data challenges. Similarly, implementation of the model requires technical capacity to be able to perform the analysis. These challenges can be more evident once the 4 country pilots have been carried out. In addition, the model focused on quantifiable criteria that can be measured in a numerical format. As a result, some policy concerns regarding the introduction of new vaccines such as equitable access or domestic vaccine production capacity, cannot be easily incorporated in a quantitative model as shown in the case study. Lastly, inputs from a limited number of respondents were included and only the top 5 decision-making criteria was collected, for example representatives from the public and/or civil society were absent from the first meeting. However, this is an exploratory study and to this end the study was not designed to reach all relevant stakeholders in the Thai healthcare system for eliciting the decision criteria. The analysis applied equal weights across the TSE criteria for simplicity due to the non-representativeness of the stakeholder participation in the meeting. The readers should keep in mind the potential impact of the above limitations on the study findings.

## Conclusion

Findings from the Thailand pilot indicate that TSE may be a beneficial approach for LMICs that have not yet developed strong and accountable decision-making processes and governance structures for immunization policy. TSE has the potential to provide a well-reasoned and systematic approach for these countries, as a starting block for structured and transparent decision-making for vaccine product selection. Furthermore, TSE could be of value to a broader set of countries, including Thailand, for aligning prioritization mechanisms and thus as a foundation to help streamline vaccine policy processes.

## Acknowledgements

This study was funded by Bill and Melinda Gates Foundation. HITAP is funded by the Thailand Research Fund under the senior research scholar on Health Technology Assessment [grant numbers RTA5980011] and MOPH, Thailand. HITAP is part of the International Decision Support Initiative (https://www.idsihealth.org/), which supports countries to get the best value for money from health spending. International Decision Support Initiative receives funding support from the Bill & Melinda Gates Foundation, the UK Department for International Development, and the Rockefeller Foundation. The findings, interpretations and conclusions expressed in this article do not necessarily reflect the views of the aforementioned funding agencies. The authors would like to thank Ms. Manushi Sharma and Mr. Md Rajibul Islam for their contribution to this project as co-investigators. The views expressed are theirs and do not necessarily represent the views of the WHO.

## Ethical issues

The method of this study was to analyse the secondary data, and did not involve the use of patient data. Therefore, ethical approval is not required.

## Competing interests

SB is a WHO consultant and RH is a WHO staff member.

## Authors’ contributions

All authors attest they meet the ICMJE criteria for authorship. WR, YT, JL, SB, BG, and RCWH contributed to conception and design of the study. WR, YT, and RAA conducted the review and analysis of comparing TSE approach with Thailand’s processes. RK and JL conducted the TSE model (pilot study in Thailand). WR, RK, and YT interpreted the results. WR, RK, and YT drafted the manuscript. All authors revised the manuscript and approved the final version. YT, BG, and RCWH provided supervision to the overall of the TSE pilot study in Thailand.

## Authors’ affiliations

^1^Health Intervention and Technology Assessment Program (HITAP), Ministry of Public Health, Nonthaburi, Thailand. ^2^Saw Swee Hock School of Public Health, National University of Singapore, Singapore, Singapore. ^3^Asc Academics, Groningen, The Netherlands. ^4^World Health Organization (WHO), Genève, Switzerland.

## Supplementary files


Supplementary file 1 contains Tables S1-S3.

Supplementary file 2 shows the detailed methodology for estimating the vaccine performance based on decision criteria.

## Key Messages

**Implications for policy makers**

- Similar to other low- and middle-income countries (LMICs), Thailand is facing financial and non-financial challenges when introducing new, high cost vaccines into their national vaccination program and this creates a greater need for robust and evidence-based mechanisms when setting vaccine priorities.
- Since introducing universal health coverage (UHC) in 2002, the Thai government has integrated its vaccination program to be part of UHC scheme. This led to the creation of a new mechanism for vaccine selection and prioritisation which can bring about misalignment between the 2 national decision-making bodies responsible for vaccine introduction.
- The newly proposed Total System Effectiveness (TSE) uses a multi-criteria decision analysis (MCDA) approach that may be useful for promoting evidence-based and demand driven vaccine selection and also help streamline different decision-making processes.

**Implications for the public**

Total System Effectiveness (TSE) was piloted in Thailand, which has established governance structures and accountable decision-making processes for immunization policy, to explore the potential use of TSE in assisting national policy-makers with prioritizing vaccines. The findings highlighted that TSE can be incorporated with existing country policy processes, and help in promoting evidence-based and demand driven vaccine selection. In addition, TSE may be able to help reconcile concerns from different decision-making bodies in a country, as is the case in Thailand, where 2 committees are responsible for vaccine policy. Lessons learned from Thailand may be beneficial to other low- and middle-income countries (LMICs) looking to use the TSE approach to develop a systematic and transparent decision-making process for immunisation policy.
