# Supplementary material for: Comparing 3 Approaches for Making Vaccine Adoption Decisions in Thailand
Source: Int J Health Policy Manag. 2020 Jan 20;9(10):439–47. doi: 10.15171/ijhpm.2020.01 (PMC7719214; doi:10.15171/ijhpm.2020.01)
Supplement: Supplementary file 1 — contains Tables S1-S3. [file ijhpm-9-439-s001.pdf]

Supplementary file 1

Table S1. Input Variables Required for Populating the TSE Model

**Variables**	**Description**
Size of target population	Size of birth cohort
Coverage of existing vaccines for different population segments	Coverage of existing BCG/DTP1-3 vaccines in Thailand by wealth quintiles and administrative regions
Vaccine schedules with which rotavirus is expected to be administered	Schedule of BCG and DTP 1,2,3 vaccine programs
Mortality rates	Background mortality, infant mortality and neonatal mortality
Disease incidence and related statistics	Incidence of rotavirus infection by severity levels and related mortality
Use of healthcare services	Distribution of inpatient and outpatient visits by healthcare setting - primary, secondary, tertiary and healthcare centres
Healthcare costs	Healthcare costs for outpatient and inpatient cases by health care setting – primary, secondary, tertiary and healthcare centres
Adverse events incidence	Background intussusception rate per 100,000
Vaccine storage and delivery	Thailand specific costs for vaccine transport and storage

Abbreviations: DTP, a combined vaccine against diphtheria, tetanus, and pertussis; BCG, Bacille Calmette Guerin vaccine.

Table S2. Characteristics of the 5 Hypothetical Rotavirus Vaccine Products

Vaccine characteristics	Rotavirus vaccine products*				
	**RVV-1**	**RVV-2**	**RVV-3**	**RVV-4**	**RVV-5**
Number of doses needed	3	2	1	3	3
Vaccine efficacy	50%	50%	65%	60%	72%
Duration of protection (weeks)**	52	52	52	78	52
Dosing schedule of first dose	DTP-1	DTP-1	DTP-1	BCG	DTP-1
Price per dose (USD)	3.6	2.2	5	8.2	6.1
Doses per vial	1	1	1	2	1
Volume of the vaccine per dose (cm^3)	46.3	17.6	24.3	22.1	34.2
Does it need a diluent?	Yes	Yes	No	No	Yes
Volume of diluent/ other component (cm^3)	45	17.6	-	-	42
Is an injection syringe/ applicator required?	No	Yes	No	No	Yes
Volume of injection syringe (cm^3)	-	2	-	-	12
Price of injection syringe/ applicator (USD)	-	0.04	-	-	0.02
Is a reconstitution syringe/adapter required?	No	No	No	No	Yes
Volume of reconstitution syringe/adapter (cm^3)	-	-	-	-	21
Price of reconstitution syringe/ applicator	-	-	-	-	0.01
Method of cooling, Vaccine					
National level	Cold chain	Cold chain	CTC	Cold chain	Freezer
Regional level	Cold chain	Cold chain	CTC	Cold chain	Freezer

Vaccine characteristics	Rotavirus vaccine products*				
	**RVV-1**	**RVV-2**	**RVV-3**	**RVV-4**	**RVV-5**
District level	Cold chain	Cold chain	CTC	Cold chain	Freezer
Health centre level	Cold chain	Cold chain	CTC	Cold chain	Freezer
Method of cooling, Diluent					
National level	Cold chain	Cold chain	-	-	Freezer
Regional level	Cold chain	Cold chain	-	-	Freezer
District level	Cold chain	Cold chain	-	-	Freezer
Health centre level	Cold chain	Cold chain	-	-	Freezer
Relative risk of a intussusception adverse event in the 1-7 days risk period					
Dose 1	6	4.2	3	2	3
Dose 2	3	2	-	1	1
Dose 3	1.7	-	-	1	1
Relative risk of a intussusception adverse event in the 8-21 days risk period					
Dose 1	1.5	1.4	0	1.2	1.1
Dose 2	1	1	-	1	1
Dose 3	1	-	-	1	1
Wastage	5%	5%	5%	5%	5%
Buffer vaccines	10%	10%	10%	10%	10%
*All 5 vaccine products considered are hypothetical					
**The mean duration of protection offered by the vaccine product					
Abbreviations: cm^3, cubic centimetre; CTC, controlled temperature chain; DTP, a combined vaccine against diphtheria, tetanus, and pertussis; BCG, Bacille Calmette Guerin vaccine; RVV, rotavirus vaccine; USD, United States Dollar.					

Table S3. Scores and ranks for the 5 hypothetical rotavirus vaccine products using the TSE approach

**Absolute scores**						
**Decision criteria**		RVV1	RVV2	RVV3	RVV4	RVV5
Safety		0	42	77	83	74
Health impact		17	17	23	34	24
Delivery costs		7	79	98	65	0
Cost-effectiveness		0	61	27	0	0
Budget impact		54	85	87	0	26
**Weighted scores**						
**Decision criteria**	**Weights**	RVV1	RVV2	RVV3	RVV4	RVV5
Safety	20%	0	8	15	17	15
Health impact	20%	3	3	5	7	5
Delivery costs	20%	8	17	20	15	0
Cost-effectiveness	20%	0	12	15	0	0
Budget impact	20%	11	17	17	0	5
Aggregate weighted score		22	58	72	39	25
**Vaccine ranking**		**5th**	**2nd**	**1st**	**3rd**	**4th**

RVV, rotavirus vaccine
