# Supplementary material for: Comparing 3 Approaches for Making Vaccine Adoption Decisions in Thailand
Source: Int J Health Policy Manag. 2020 Jan 20;9(10):439–47. doi: 10.15171/ijhpm.2020.01 (PMC7719214; doi:10.15171/ijhpm.2020.01)
Supplement: Supplementary file 2 — shows the detailed methodology for estimating the vaccine performance based on decision criteria. [file ijhpm-9-439-s002.pdf]

Supplementary file 2. The Detailed Methodology for Estimating the Vaccine Performance Based on Decision Criteria

The performance of using the rotavirus vaccine (RVV) products in nation-wide RVV vaccination program is estimated based on 5 decision criteria. They have been mentioned below with an explanation of their outcome measures, namely:

1) Safety
2) Health impact
3) Delivery costs
4) Cost-effectiveness
5) Budget impact

These 5 decision criteria mentioned above, were measured based on the methodology used in already validated and published models like UNIVAC[1], the Vaccine-Technology Impact Assessment (V-TIA) tool[2] and published guidelines for budget impact analysis and cost-effectiveness analysis[3,4]. Locally relevant input parameters were used to generate results relevant for the Thailand healthcare system. Detailed explanation of the methodology for each performance characteristic can be found below.

3.1. Safety

Outcome measure: Number of intussusception cases

Input parameters

- Intussusception incidence per year
- Relative risk of intussusception following a RVV dose (different risk assumed for first week after vaccination and the second & third week)

Outcome estimation

The method for estimating the number of intussusception cases was based on the previously published model UNIVAC. Relative risk of intussusception among vaccinated infants in comparison to non-vaccinated infants was used to calculate the number of intussusception cases expected for the vaccination program implemented using each of the 5 different RVV products. The intussusception incidence per year was converted to a weekly incidence. This allowed for the estimation of the number of intussusception cases when no RVV was used. The number of intussusception cases per week was then multiplied by the relative risk of intussusception

following a RVV administration to estimate the number intussusception cases in the vaccinated children. Different relative risk values were assumed for the first week following vaccine administration and for the following two weeks as the relative risk of intussusception is much higher initially in the first week than the following two weeks. After 3 weeks it was assumed that the relative risk of intussusception among the vaccinated and non-vaccinated children is the same.

3.2. Health Impact

Outcome measure: Number of rotavirus cases averted

Input parameters

- Size of birth cohort
- Neonatal morality
- Infant mortality
- Under 5-year old mortality
- Incidence of rotavirus infections in the under 5-year olds
- Distribution of rotavirus cases in the under 5-year old population
- Vaccination schedule (moment of immunization)
- Vaccine efficacy
- Vaccination coverage

Outcome estimation

The method for estimating the number of rotavirus cases, the distribution of the rotavirus cases and the rotavirus cases averted are based on the previously published model UNIVAC. The model simulated a birth cohort from 0 years, until the children reach the age of 5. The incidence of rotavirus infection is known in the under 5-year old population. Therefore, the number of life years lived by the birth cohort in the 5-year timeframe was calculated by adjusting for the neonatal, infants and under 5-year old mortality. The life years lived by children in the cohort was then multiplied by the under 5-year rotavirus incidence, to obtain the total number of rotavirus cases in the 5-year timeframe.

As rotavirus infection incidence is not evenly distributed over the time from birth until the age of 5, a log-logistic distribution was applied to the cases to redistribute the cases over the birth cohort's first 5 year of life. The scale and shape values for the log-logistic distribution were 2.25 and 64.64, respectively based on the estimates from Hasso-Agopsowicz et al., 2019[5].

The impact of RVV also dependent on the moment of vaccination, vaccine coverage, vaccine efficacy and the duration of protection of the vaccine. The RVV simulated in the model were co-administered as per the schedule of one or more of the existing vaccines, as specified in vaccine product characteristics table. Existing vaccine included BCG, DTP-1, DTP-2 and/ or DTP-,3 recommended in first, sixth, tenth and fourteenth week of life, respectively.

The first dose of a RVV could either be administered alongside BCG or DTP-1. The schedule of the first dose could have an impact as BCG is given in the first week and DTP-1 in the sixth week after birth and the risk of developing rotavirus infection is significantly high for the infants in the first few weeks after birth. The coverage rates of the RVV was assumed to be similar to the coverage observed for the existing vaccines along with which the RVV was assumed to be co-administered. The vaccine efficacy of the RVV was assumed to be reached when a child received the full course of the vaccine. When a child receives only half of the total doses, the efficacy was also halved to correct for a lower protection against infection. The duration of protection was simulated using the mean duration of protection (1 - a normal cumulative distribution of the average duration of protection) and the standard deviation of the average duration of protection. When a child does not receive the full course of a RVV, the duration of protection was set to be the same as when a child would receive all doses, but with lower protection against infection. The duration of protection was simulated from the time at which the child receives the last dose of the vaccine course.

3.3. Delivery costs

Outcome measure: Transport costs, storage costs and healthcare worker salary/training costs

3.3.1. Transport cost

This included the cost of transporting the vaccine from the national level storage to the district storages and health centre facilities. For Thailand, the cost of transportation was only considered for the transport from district level to health facilities. The initial cost of transport from national to district level facilities usually are included in the commodity price itself.

Input parameters

- Cooling requirements per vaccine
- Volume per vaccine
- Petrol price
- Thailand’s surface area

- Number of vaccines needed
- Number of stock replenishments per level of the supply chain

Outcome estimation

The V-TIA tool provided transport costs per cm3 per km for products cooled between 2 and 8 °C and an estimate for the distance between the levels of the supply chain. The distances between a level of the supply chain and the subsequent level was estimated using the Thailand's surface area with Tanzania as a reference country.

$$Distance\ between\ level\ x\ and\ y\ in\ Thailand = \sqrt{(\text{Thailand's surface area/ Tanzania area})} \times \text{ distance between level x and y in Tanzania}$$

For Thailand, the transport costs were only estimated for transport between the lower two levels of the supply chain (ie, transport to district level and health facilities) as the costs of transport from the national storage is already taken into account in the vaccine procurement process.

The transport costs provided by the V-TIA tool can be subdivided into costs for transport and costs for carriers used during transport. The V-TIA tool estimates the costs per cm^3 per km based on the petrol price. Other costs included in this estimate are maintenance (15% of the petrol price), depreciation cost per km, fuel consumption of the assumed vehicle used for transport. Multiplying the distance of the transport between the levels of the supply chain by the costs per cm3 per km and by the volume of the vaccines in cm3 results in calculation of costs of the vaccine for transport from the national to the lowest level of the supply chain.

The V-TIA tool also provided costs for the vaccine carriers. Beside the total volume of the vaccines, the number of stock replenishments determine the estimated number needed of these carriers as multiple stock replenishments require less carriers than a single annual stock replenishment. The costs of the carriers were annualized as it was assumed that these carriers could be used across the economic life of the carriers. The V-TIA tool provides the costs of the carriers used for cold chain conditions. The vaccines in the model could have 3 cooling specifications: cold chain, controlled temperature chain (CTC) and freezer storage. The assumption was made that carriers for frozen transport were twice as expensive as the cold chain carriers and that CTC vaccines did not need specific carriers.

3.3.2. Storage costs

This included the cost of storing the vaccine at the district storages and health centre facilities as per the temperature requirements of the different vaccine products. In Thailand, no additional expense was considered for storage capacity as there are existing storage capacities which can be used.

3.3.3. Healthcare worker costs

As Thailand healthcare system has sufficient healthcare workers/ vaccinators, no additional healthcare workers were expected to be recruited for RVV campaigns. Thus, no additional expense as salaries for additional healthcare workers recruited specially for RVV campaigns was accounted for. However, costs required for conducting trainings of healthcare workers at the national, sub-national (provincial) and district level was calculated.

Input parameters

- Cost of conducting training workshop at the national level
- Number of national-level workshops
- Cost of conducting training workshop at the provincial level costs
- Number of provincial workshops
- Cost of conducting training workshops at the district level costs
- Number of district level workshops

Outcome estimation

A new vaccination program needs training of those involved. In the model 3 types of training workshops are taken into account, ie, trainings workshops at the national, provincial and district level. For the Thailand setting, one national workshop happens every two years and one workshop happens annually at the provincial level. As the model is unable to accommodate a biennial workshop, we assumed 1 training workshop to happen annually at the national level, and included half of the involved costs of the biennial workshop. At the district level, the number of training workshops is dependent on the number of vaccinators needed. The total number of vaccinators needed for the vaccination program was estimated and the number of training workshops required at the district level calculated by assuming a maximum of ten participants to be attending one training. The number of vaccinators was calculated based on the time needed to administer one vaccination, the number of doses required for the vaccine product and, and the hours a vaccinator works per week. The time needed for a vaccination was set at 1 minutes and a fulltime vaccinator

was assumed to work 36 hours a week and 46 weeks per year. Multiplying the doses administered by the time needed for one vaccination results in the total time needed for administration of a vaccine to a child. This result is divided by the total time a vaccinator can vaccinated per year and gives the number of vaccinators needed.

3.4. Cost-effectiveness analysis

Outcome measure: Incremental cost-effectiveness ratio of implementing a national vaccination program for RVV in comparison to no vaccination program (USD/DALY averted)

Input parameters

- Percentage severe cases of all rotavirus cases
- Outpatient visit rate per case
- Hospitalization rate of severe rotavirus cases
- Mortality rate rotavirus case
- Average outpatient costs
- Average inpatient/ hospitalization costs
- DTP-1 coverage
- Life-expectancy per age
- Discount rates for costs and health outcomes
- DALY lost per non-severe, severe and intussusception case

Outcome estimation

Cost-effectiveness analysis calculated the cost per DALY averted. For the cost component, it accounted for the program costs and cost-savings arising from averted cases. For the health impact, life expectancy, DALYs lost due to healthy time lost due to the illness by the severity of the rotavirus infection case, average duration of illness and the side effect of intussusception or premature mortality were calculated.

The program costs included the cost of purchasing the commodity (vaccine), the transport costs, and costs of conducting training for healthcare workers, and cost of intussusception, as computed in the abovementioned criteria. The commodity costs are estimated by multiplying the needed vaccines by the cost per vaccine, whereby the number of vaccines is adjusted to account for wastage and to provide a buffer at the health facilities to prevent that vaccines are not in stock when children need their vaccination. Delivery costs are already discussed above. The

intussusception costs are made as it was assumed that children suffering from this condition would be hospitalized.

The computation of the rotavirus infection treatment costs were based on the utilization of healthcare services. Therefore, the rotavirus cases were disaggregated by severity levels, whereby a severe case is defined as a case that requires hospitalization. The model simulated the rotavirus cases over a 5-year time period. Per year the number of rotavirus cases were estimated and disaggregated in outpatient visits and inpatient visits based on the severity levels. Combining the number of these visits with the associated costs evaluates the total rotavirus related treatment costs. The cost-effectiveness was estimated comparing a vaccination program to no-vaccination program. Savings of a vaccination program were reflected in direct medical costs averted and health benefits included DALYs averted because of the rotavirus infection cases avoided post vaccination. DALYs losses were taken into account for a non-severe rotavirus case, severe RVV case, intussusception case and related death. The DALY losses associated with death were estimated using the life expectancy at the age of death.

3.5. Budget impact

Outcome measure: 5-year budget impact of implementing the RVV program on the healthcare budget

Input parameters

- Program costs (Commodity costs, storage costs, transport costs, and training costs)
- Cost savings due to averted disease

Outcome estimation

The financial impact a vaccination program against rotavirus infection was assessed in a budget impact analysis from the Ministry of Health perspective, with a time horizon of 5 years[3,4]. All the birth cohorts in the 5-year time horizon were included in the analysis and the cost and benefits were estimated for the first until the fifth year or the end of the time-horizon, whichever is earlier. The program costs for implementing the vaccination program for the 5-birth cohorts and cost savings arising from RVV infection cases avoided from 0-5 years of age due to vaccination were accounted. The vaccine coverage of BCG and DTP-1 to 3 was assumed to increase slightly per year by a fixed percentage. Projections of the size of future birth cohorts were also taken into

account. Per year new vaccinators were assumed to be trained as it was assumed that 0.1% of the vaccinator workforce needed to be replaced every year.

Reference

1. Anwari P, Debellut F, Pecenka C, et al. Potential impact and cost-effectiveness of rotavirus vaccination in Afghanistan. *Vaccine*. 2018;36(51):7769-74.
2. Giersing BK, Kahn AL, Jarrahian C, et al. Challenges of vaccine presentation and delivery: How can we design vaccines to have optimal programmatic impact?. *Vaccine*. 2017;35(49):6793-7.
3. Chaikledkaew U, Kittrongsiri K. Guidelines for health technology assessment in Thailand --the development process. *J Med Assoc Thai*. 2014;97:S4-9.
4. Sullivan SD, Mauskopf JA, Augustovski F, et al. Budget impact analysis—principles of good practice: report of the ISPOR 2012 Budget Impact Analysis Good Practice II Task Force. *Value health*. 2014;17(1):5-14.
5. Hasso-Agopsowicz M, Ladva CN, Lopman B, et al. Global review of the age distribution of rotavirus disease in children aged< 5 years before the introduction of rotavirus vaccination. *Clin Infect Dis*. 2019;69(6):1071-8.
